# Chimeric Viruses Enable Study of Antibody Responses to Human Rotaviruses in Mice

**DOI:** 10.3390/v16071145

**Published:** 2024-07-16

**Authors:** Sarah Woodyear, Tawny L. Chandler, Takahiro Kawagishi, Tom M. Lonergan, Vanshika A. Patel, Caitlin A. Williams, Sallie R. Permar, Siyuan Ding, Sarah L. Caddy

**Affiliations:** 1 Baker Institute for Animal Health, Cornell University, Ithaca, NY 14850, USA; sarahwoodyear@cornell.edu (S.W.);; 2 Department of Molecular Microbiology, Washington University in St. Louis, St. Louis, MO 63101, USA; 3 Department of Pediatrics, Weill Cornell Medicine, New York, NY 10001, USA

**Keywords:** rotavirus, antibody, reverse genetics, vaccine

## Abstract

The leading cause of gastroenteritis in children under the age of five is rotavirus infection, accounting for 37% of diarrhoeal deaths in infants and young children globally. Oral rotavirus vaccines have been widely incorporated into national immunisation programs, but whilst these vaccines have excellent efficacy in high-income countries, they protect less than 50% of vaccinated individuals in low- and middle-income countries. In order to facilitate the development of improved vaccine strategies, a greater understanding of the immune response to existing vaccines is urgently needed. However, the use of mouse models to study immune responses to human rotavirus strains is currently limited as rotaviruses are highly species-specific and replication of human rotaviruses is minimal in mice. To enable characterisation of immune responses to human rotavirus in mice, we have generated chimeric viruses that combat the issue of rotavirus host range restriction. Using reverse genetics, the rotavirus outer capsid proteins (VP4 and VP7) from either human or murine rotavirus strains were encoded in a murine rotavirus backbone. Neonatal mice were infected with chimeric viruses and monitored daily for development of diarrhoea. Stool samples were collected to quantify viral shedding, and antibody responses were comprehensively evaluated. We demonstrated that chimeric rotaviruses were able to efficiently replicate in mice. Moreover, the chimeric rotavirus containing human rotavirus outer capsid proteins elicited a robust antibody response to human rotavirus antigens, whilst the control chimeric murine rotavirus did not. This chimeric human rotavirus therefore provides a new strategy for studying human-rotavirus-specific immunity to the outer capsid, and could be used to investigate factors causing variability in rotavirus vaccine efficacy. This small animal platform therefore has the potential to test the efficacy of new vaccines and antibody-based therapeutics.

## 1. Introduction

Rotavirus vaccines have been highly successful at reducing the burden of rotavirus-induced gastroenteritis in children in high-income countries. Two oral vaccines were first approved in 2006 and 2008, developed from a human rotavirus strain (Rotarix, GSK, Rixensart, Belgium) and a human–bovine rotavirus reassortant virus (Rotateq, Merck & Co., Rahway, NJ, USA) [1,2]. A total of four live attenuated rotavirus vaccines are now pre-qualified with the World Health Organization, and a further two are country-specific [3]. However, live attenuated vaccines have failed to protect infants in low- to middle-income countries, with vaccine efficacy often lower than 50% [4,5], leading to higher rates of gastroenteritis deaths in unprotected infants. Consequently, there is a pressing need for the development of improved vaccines.

A major hurdle for vaccine development has been the absence of suitable pre-clinical models to test new vaccine candidates. Rotavirus strains are highly species-specific, meaning that human rotavirus strains are primarily pathogenic in humans, and murine rotavirus strains only cause disease in mice [6,7]. This hinders the study of disease pathology and immune responses to human rotavirus strains in mice, as human rotavirus strains only replicate to low titres and cause minimal disease in this species.

In this study, we aimed to develop a new strategy to study the immune response to human rotaviruses in a mouse model. To achieve this, we generated a chimeric rotavirus strain that can replicate well in mice and contains key immunogenic proteins from a human rotavirus strain. This approach builds on previous work that showed reassortment of certain murine rotavirus genes with those from a non-murine rotavirus strain could still permit virus replication in mice [6].

The advent of rotavirus reverse genetics has provided the field with the capacity to rapidly generate chimeric rotaviruses with relative ease [8,9]. Rotaviruses have a triple-layered structure with an outer capsid composed of two proteins, VP4 and VP7, which become a major target of the adaptive immune response [10]. Chimeric rotaviruses have previously been produced using reverse genetics with a murine backbone and human rotavirus VP4 protein [11], and we have now extended this by successfully generating a chimeric rotavirus with VP4 and VP7 from a human rotavirus strain. As a control, we also generated a chimeric virus encoding the outer capsid proteins of a heterologous murine rotavirus strain. We demonstrated that both viruses could infect neonatal mice and replicate to comparable titres.

Antibody responses to human rotavirus proteins in chimeric rotaviruses have not previously been studied in mice. We aimed to determine the magnitude and specificity of the antibody response to human rotavirus outer capsid proteins using our small animal model. We observed strong germinal centre formation in the draining lymph nodes of mice infected with both chimeric viruses, which correlated with antibody production. Neutralisation and ELISpot assays were used to clearly demonstrate that the chimeric virus with human rotavirus outer capsid proteins induced antibodies with human rotavirus specificity. This novel approach to studying human rotaviruses in a small animal model will be valuable for pre-clinical evaluation of vaccine efficacy and therapeutics targeting the outer capsid of human rotaviruses.

## 2. Materials and Methods

### 2.1. Cells and Viruses

MA104 African green monkey kidney cells, provided by Dr. John Parker (Baker Institute for Animal Health, Cornell University, Ithaca, NY, USA), were grown in Dulbecco’s Minimum Essential Media (DMEM) supplemented with 10% heat-inactivated fetal bovine serum, 100 IU/mL penicillin, and 100 μg/mL streptomycin (complete DMEM). Reagent details are listed in Table 1.

The pre-existing rotavirus strains used in this study were the Rotarix vaccine strain (G1P [8]) and the primate strain SA11 (G3P [2]). Two rotavirus chimeric strains were developed using a published plasmid-based reverse genetics system [12]. Using the reassortant virus rD6/2-2g as the backbone, chimeric viruses were rescued with a human CDC-9 strain VP4 and VP7 (human outer capsid proteins), or a murine strain (ETD) VP4 and VP7, validated by sequencing. All viruses were propagated in MA104 cells following activation with 10 μg/mL TPCK-treated trypsin at 37 °C for 30 min. Prior to in vivo infection, viruses were diluted to the appropriate titre in sterile PBS without calcium chloride and magnesium chloride.

### 2.2. Virus Quantification by Fluorescent Focus Assay (FFA)

A fluorescent focus assay was used to determine viral titre (in fluorescent focus units, FFU) as previously described [13]. Briefly, MA104 cells were infected with the virus for 16 h, then cells were fixed with 1:1 methanol–acetone at −20 °C for 20 min. After blocking with PBS-2%FBS for 20 min at room temperature, 10 μg/mL rotavirus polyclonal antibody (sheep) diluted in PBS-2%FBS was added for 1 h at room temperature. After three washes with PBS supplemented with 0.1% Tween-20 (PBS-T), 4 μg/mL Alexa Fluor 488 Donkey Anti-Sheep IgG and Hoechst 33,342 diluted in PBS-2%FBS were added to each well, and the plate was incubated for 1 h at room temperature. All plates were coated with PBS and kept at 4–6 °C prior to imaging. Quantification of rotavirus-infected cells was achieved using the BioTek Cytation 7 Cell Imaging Multimode Reader and Gen5 Image Prime (v3.13) software.

### 2.3. Rotavirus Infection of Mice

129S6/SvEvTac mice (Taconic Biosciences, Germantown, NY, USA) were maintained by an in-house breeding colony housed at the Baker Institute for Animal Health. All mouse work was approved by the Cornell University Institutional Animal Care and Use Committee (IACUC), Protocol 2022-0152. Seven-day-old pups were infected with 1 × 10^4^ FFU of virus by oral gavage. Pups were monitored for the development of diarrhoea, and scored positive if mucus or liquid stool was observed. Stool samples were collected once daily post-infection from each litter, then pooled and diluted 1:10 in PBS. Diluted stool was centrifuged at 8000× *g* for 5 min to remove debris, and the supernatant was stored at −80 °C. Blood samples were collected from the lateral saphenous vein at 4, 6, and 8 weeks old, and by terminal cardiac puncture at 10 weeks of age. All blood samples were centrifuged at 6000× *g* for 5 min, and sera were stored short-term at 4 °C. In separate experiments, 14-days-post-infection mice were humanely culled with terminal cardiac puncture samples collected, and the Peyer’s patches (PPs), mesenteric lymph nodes (MLNs), and spleens were harvested.

### 2.4. Quantification of Virus Shedding by RT-qPCR

RNA extraction from a clarified stool suspension was achieved using the Monarch Total RNA Miniprep Kit according to manufacturer’s instructions. Each sample was eluted in a total volume of 50 μL nuclease-free water, followed by denaturation of dsRNA at 95 °C for 5 min. RT-qPCR was performed using the Luna Universal One-Step RT-qPCR Kit per the manufacturer’s instructions, with 5 μL of RNA in a total reaction volume of 20 μL using NSP5 forward primer CTGCTTCAAACGATCCACTCAC at 400 nM, NSP5 reverse primer TGAATCCATAGACACGCC at 400 nM, and NSP5 TaqMan probe FAM-TCAAATGCAGTTAAGACAAATGCAGACGCT-TAMRA at 200 nM. The reaction was carried out on a QuantStudio 3 thermocycler (Applied Biosystems, Waltham, MA, USA) under the cycling conditions of 55 °C for 10 min, 95 °C for 1 min, and 40 cycles of 95 °C for 10 s and 60 °C for 30 s. A 10-fold serial dilution of SA11 total RNA was included on each plate to quantify rotavirus genome copies per mL of stool supernatant using QuantStudio Design & Analysis Software (v1.5.1). A lower limit of quantification of 100 genome copy numbers was set and assigned to samples with no detectable virus.

### 2.5. ELISAs

ELISAs were performed to detect IgG-specific anti-rotavirus antibodies using an in-house method as previously described [13]. MA104 cells were infected with rotavirus to produce infected cell lysate, or phosphate-buffered saline (PBS) to produce control cell lysate. Cells were collected and resuspended in Radio-Immunoprecipitation Assay (RIPA) buffer supplemented with protease inhibitors. The Bicinchoninic Acid Kit for protein determination was used to measure protein concentration following the manufacturer’s instructions, and lysate stocks were diluted to 1 mg/mL in PBS.

Plates were washed three times using PBS-T between each step. High-binding 96-well plates (Greiner Bio-One, Monroe, NC, USA) were coated with 5 μg/mL rotavirus-specific polyclonal antibody (sheep) in PBS and incubated at 4–6 °C for 16 h. Plates were then blocked with 5% milk–PBS-T at room temperature for 1 h. The purified cell culture lysates (Rotarix- and SA11 virus-infected lysate or mock-infected control lysate) were diluted to 10 μg/mL in PBS and incubated at 37 °C for two hours. Sera were diluted 1:200 in 5% milk–PBS-T and added in duplicate and incubated at 37 °C for 2 h. Positive and negative control sera from known infected and uninfected mice were included on each plate. The anti-mouse IgG HRP secondary antibody was diluted 1:1000 in 5% milk–PBS-T and added before plates were incubated at 37 °C for 1 h. To detect the bound antibody, 3,3’,5,5’-tetramethylbenzidine (TMB) was incubated at room temperature for 10 min. The reaction was stopped with 1M sulfuric acid (H_2_SO_4_), and the optical density (OD) was read at 450 nm using the BioTek Cytation 7 Cell Imaging Multimode Reader. The OD was normalised by subtracting the OD of the mock-infected control cell lysate well from the OD of the virus-infected cell lysate well.

### 2.6. Western Blot

Rotarix- and SA11-infected cell lysates were denatured in 4× Laemli buffer at 95 °C for 5 min, then separated on a 4–15% Mini-PROTEAN TGX gel (Bio-Rad, CAT#456-1083). Proteins were transferred to a polyvinylidene difluoride (PVDF) membrane by a Trans-blot Turbo Transfer System (Bio-Rad, Hercules, CA, USA). The membrane was blocked with 5% milk–PBS-T at room temperature for 1 h, then incubated with sera from mice infected with either human outer capsid rotavirus or murine outer capsid rotavirus diluted 1:250 in 5% milk–PBS-T for 12 h at 4–6 °C. Following three washes in PBS-T, the membrane was incubated with anti-mouse IgG HRP diluted 1:500 in 5% milk–PBS-T for 1 h at room temperature. Blots were washed three times in PBS-T and visualised using clarity Western ECL substrate and a ChemiDoc MP imaging system.

### 2.7. Extracellular Neutralisation Assay

MA104 cells were seeded in a 96-well black-sided plate (Corning 3340) at 2 × 10^4^ per well in complete DMEM and incubated at 37 °C for 4 h to allow cells to adhere. One 1:10 dilution of serum in serum-free media (SFM) was incubated with trypsin-activated rotavirus at 37 °C for 1 h. The serum–virus mixture was then added in triplicate to seeded cells. After 1 h at 37 °C, 50 μL complete DMEM was added to each well and the plate was incubated at 37 °C for 16 h. Rotavirus neutralisation was quantified by FFA.

### 2.8. Intracellular Neutralisation Assay

A previously published intracellular neutralisation assay protocol was applied to sera from mice infected with the human or mouse outer capsid chimeric viruses [9]. Using a Neon Transfection System Kit, sera were diluted 1:3 in PBS and mixed with 2 × 10^5^ MA104 cells suspended in Resuspension Buffer R. Sera were then electroporated with two pulses at 1400 V and a 20-pulse width using the Neon^®^ Transfection System (Thermo Fisher Scientific, Waltham, MA, USA). Electroporated cells were resuspended in complete DMEM and plated onto a 96-well black-sided plate in duplicate. After incubation at 37 °C for 4 h, wells were washed once with PBS, and trypsin-activated rotavirus in SFM was added to each well. Infection and virus quantification then proceeded as for the extracellular neutralisation assay, described above.

### 2.9. Isolation of Single Cells from Lymph Nodes and Spleens

Peyer’s patches (PPs), mesenteric lymph nodes (MLNs), and spleens were harvested from infected mice 14 days post-infection or control uninfected mice. The tissues were homogenised through 70 μm mesh cell strainers to obtain single-cell suspensions, and then washed with RPMI supplemented with 2%FBS (RPMI-2%FBS) by centrifugation at 300× *g* for 5 min. The PP and MLN cell pellets were resuspended in 1 mL cold staining buffer (PBS-1%FBS), then filtered through 70 μm mesh, and washed and resuspended in 100 μL staining buffer. Spleen cell pellets were resuspended in red blood cell lysis buffer and incubated at room temperature for 3 min. These were then washed with PBS by centrifugation and resuspended in RPMI-2%FBS.

### 2.10. Flow Cytometry

PP and MLN single-cell suspensions were incubated with Fc Block (1:100) in staining buffer for 30 min at 4 °C, then cells were incubated with viability dye and fluorescently conjugated antibodies targeting B220, GL7 Antigen, CD95, and CD45 in staining buffer for 30 min at 4 °C. Single-color controls were included using PP cells or compensation beads. Cells were washed twice with staining buffer by centrifugation and resuspended in 100 μL 4%PFA-PBS at 4 °C for 15 min to fix. Cells were then washed twice by centrifugation, and the cell pellets were resuspended in 300 μL staining buffer. Cells were analysed using a BD LSRFortessa X-20 (BD Biosciences, San Jose, CA, USA) and BD FACSDiva Software (v9.0), and data analysis was performed in FlowJo (v10.9.0).

### 2.11. ELISpot Assay

Spleen single-cell suspensions were analysed by ELISpot to identify rotavirus-specific B cell responses. PVDF-based membrane plates (MSIP white, Mabtech, Cincinnati, OH, USA) were activated with 35% ethanol, followed by five washes with sterile water. All further washing steps were performed five times with sterile PBS. Plates were coated with 2.7 × 10^4^ FFU Rotarix per well and incubated at 4 °C for 16 h. Wells were washed to remove excess antigen, and incubated with RPMI supplemented with 10% FBS, 100 IU/mL penicillin, and 100 μg/mL streptomycin for 30 min at room temperature. The media was removed, and 3 × 10^5^ spleen cells were incubated in triplicate at 37 °C for 16 h. The plate was then washed, and 1 μg/mL anti-mouse IgG biotin in PBS-0.5%FBS was incubated for 2 h at room temperature. After washing, 1:1000 streptavidin–ALP diluted in PBS-0.5%FBS was incubated for 1 h at room temperature. The plate was washed a final time before BCIP/NBT-plus for ALP was added and allowed to develop until distinct spots emerged. Colour development was then stopped by rinsing the plate with water, and plates were left to dry before imaging on the upright microscope of a BioTek Cytation 7 Cell Imaging Multimode Reader.

### 2.12. Statistics

Statistical analysis was performed using GraphPad Prism (v10.2.0). Immune response outcomes for two groups were analysed by unpaired two-tailed t-tests. Dependent outcomes reported for three or more groups were analysed by one-way ANOVA and pair-wise comparisons reported with Tukey’s adjustment for multiple comparisons. Outcomes reported over time were analysed by repeated measures ANOVA and pair-wise comparisons reported with Bonferroni’s correction for multiple comparisons. Statistical differences were considered significant at *p*-values < 0.05 for all comparisons. Error bars indicate the standard error of the mean.

## 3. Results

### 3.1. Construction and Characterisation of Chimeric Rotaviruses Expressing Murine or Human Strain Outer Capsid Proteins

**Figure 1:**
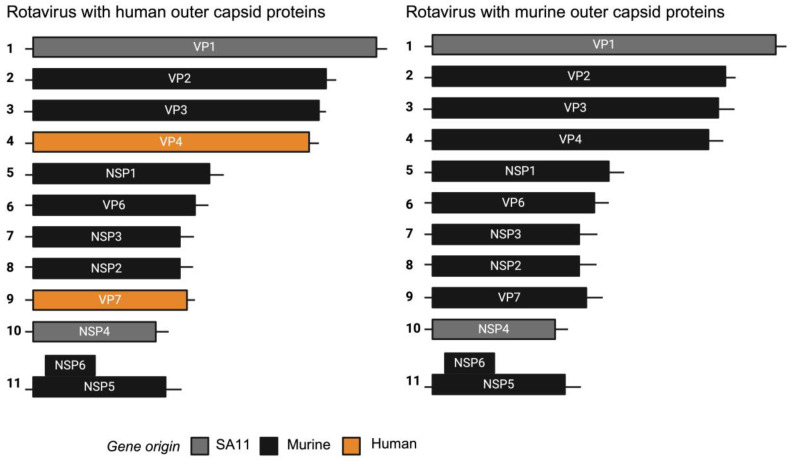
Schematic diagram of segmented dsRNA genome of chimeric rotaviruses. Reverse genetics was used to generate chimeric viruses encoding either human or murine outer capsid proteins.

We previously generated a chimeric virus named rD6/2-2g, which has a murine rotavirus backbone of the non-tissue-culture-adapted wild-type murine EW strain with gene segment 4 (VP4) of the simian rotavirus RRV strain. Gene segments 1 (VP1) and 10 (NSP4) of simian rotavirus strain SA11 were introduced to enhance rescue by reverse genetics. In addition, we generated a murine reassortant virus with gene segment 4 (encoding VP4) from the human rotavirus strain CDC9 (G1P [8]) that replicates in mice [11]. To generate a murine-like virus with an entirely human outer capsid composition, we inserted both gene segment 4 and gene segment 9 (encoding VP7) of CDC-9 into the murine-like rD6/2-2g backbone. As a control, we also produced a chimeric virus encoding the VP4 of the cell-culture-adapted murine ETD strain. This generated two different chimeric viruses that were identical except one encoded a human rotavirus outer capsid and the other a murine rotavirus outer capsid, as depicted in Figure 1.

### 3.2. Chimeric Rotaviruses with Human Outer Capsid Proteins Replicate in Mice

To examine the ability of chimeric rotaviruses to replicate and cause disease in mice, two separate litters of five pups (mouse outer capsid rotavirus) or six pups (human outer capsid rotavirus) were infected with virus by oral gavage at seven days old. For comparison with an entirely human rotavirus strain, we used Rotarix (human vaccine strain) to infect an additional litter of five pups at the same viral titre. Rotarix has an amino acid identity to CDC-9 of 98.3% for VP4 and 94.5% for VP7, so serological cross-reactivity was expected. All pups in each litter were monitored daily for the development of diarrhoea, and stool samples were collected for quantification by qPCR.

**Figure 2:**
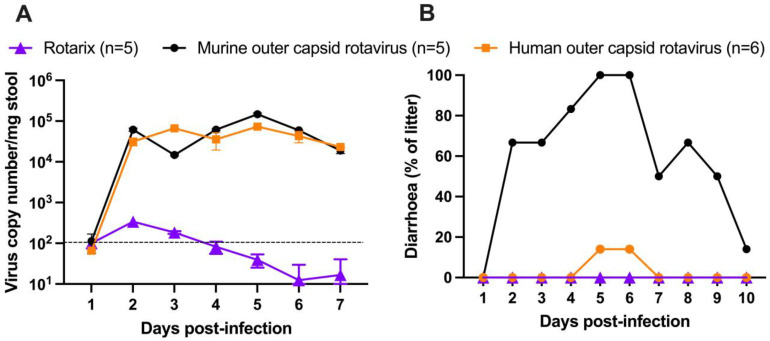
Virus shedding and clinical disease induced by oral infection of neonatal mice with chimeric rotaviruses compared to a human rotavirus strain. (**A**) Viral load detected in stool by qPCR (dotted line for lower limit of quantification). (**B**) Diarrhoea observed in neonatal mice infected at seven days old. Numbers in brackets in the key indicate the number of pups per litter.

As expected, Rotarix did not robustly replicate and was detectable at only low copy numbers in pup stool (Figure 2A). In contrast, both chimeric rotaviruses showed strong evidence of viral amplification from day 2 onwards. Viral shedding was detectable for at least seven days. As shown in Figure 2B, diarrhoea was only detected in the litter of pups infected with the chimeric virus expressing the murine rotavirus outer capsid proteins. The chimeric virus with human rotavirus outer capsid proteins was significantly attenuated in comparison. Given that the viral loads were comparable, this suggests that the outer capsid proteins are important in the pathogenesis of diarrhoea in this model, but the mechanism remains unclear.

### 3.3. Human Rotavirus-Specific Antibody Responses Are Generated in Mice Infected with Chimeric Rotaviruses

**Figure 3:**
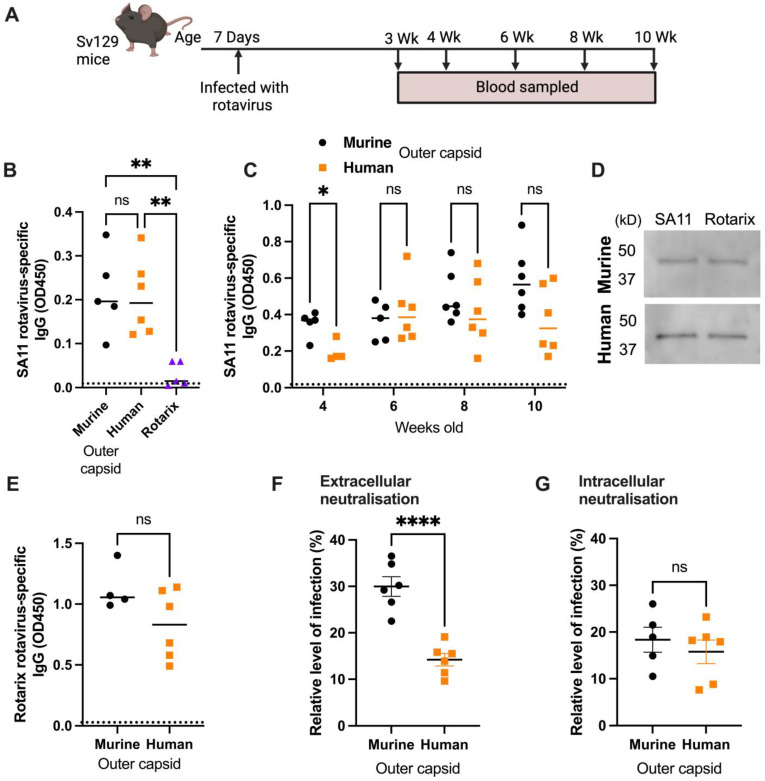
Analysis of serum antibody responses in mice infected with chimeric rotaviruses. Mice were infected with chimeric rotaviruses (murine outer capsid as black circles, human outer capsid as orange squares) or Rotarix control (shown as purple triangles) at seven days old and serum samples were collected for antibody analysis at the timepoints shown in the schematic diagram (**A**). For all graphs, each point corresponds to an individual mouse; note that some samples were not available for all assays due to limited sample volumes. (**B**) Analysis of serum from 21-day-old mice by sandwich ELISA with primate lysate. (**C**) Longitudinal samples analysed by sandwich ELISA with primate rotavirus. (**D**) Western blot of SA11- and Rotarix-infected cell lysates, probed with sera from mice infected with either murine outer capsid rotavirus or human outer capsid rotavirus. (**E**) Samples from 10-week-old mice analysed by sandwich ELISA with human rotavirus (Rotarix strain). (**F**) Extracellular neutralisation of human rotavirus by serum samples from 10-week-old mice as quantified by fluorescent focus assay. (**G**) Intracellular neutralisation of human rotavirus by serum samples from 10-week-old mice as quantified by fluorescent focus assay. Dashed horizontal lines in Figure (**A**–**C**) represent the positive threshold based on the OD450 of serum from uninfected control mice. Statistical significance was determined by one-way ANOVA (**B**), repeated measures ANOVA (**C**), or unpaired two-tailed t-tests (* *p* < 0.05; ** *p* < 0.01; **** *p* < 0.0001). Tukey’s adjusted pair-wise comparisons (**B**) and Bonferroni-corrected pair-wise comparisons (**C**) are shown.

Antibody responses following the infection of three litters of seven-day-old mice with different rotaviruses were analysed to determine the immunogenicity of the chimeric strains. Serum samples were collected from mice infected with chimeric rotaviruses at 2 weeks post-infection and compared with samples from mice infected with Rotarix. An in-house sandwich ELISA based on the SA11 primate rotavirus strain (therefore distinct from both the human and murine strains) was used to show that rotavirus-specific IgGs were readily detected in mice infected with the chimeric rotaviruses, but no antibody response was evident in mice infected with Rotarix (Figure 3B).

Next, we wanted to determine if there were any differences between longitudinal antibody responses in mice infected with either chimeric virus, so we infected two further litters of six pups each. We demonstrated that antibody responses were detected for over two months following infection (Figure 3C). Despite heterogeneity in the antibody responses within litters, there were no significant differences between IgG titres in mice infected with chimeric viruses expressing the murine or human outer capsid proteins from weeks 6 to 10.

To determine whether a sandwich ELISA could differentiate between the antibody responses of the two litters if a homologous antigen to one of the strains was used, we generated a lysate of cells infected with the Rotarix strain. We analysed samples collected at the 10-week timepoint and, as shown in Figure 3E, there was no significant difference between IgG responses detected using the Rotarix-based ELISA. This likely reflects the fact that the infected cell lysate used in the sandwich ELISA contains all rotavirus proteins, and the chimeric viruses are identical except for VP4 and VP7. This supports previous work that has shown this type of ELISA predominantly detects antibodies specific for the inner capsid protein VP6 [14], an immunodominant antigen that is identical between both chimeric virus strains. To confirm this, we performed a Western blot with the Rotarix- and SA11-infected cell lysates and probed with sera from mice infected with either the murine outer capsid rotavirus or human outer capsid rotavirus (Figure 3D). A distinct band of ~45 kD, corresponding to VP6, was detected by antibodies from both infected mice at a comparable level for either lysate.

To evaluate whether antibodies specifically targeting the human rotavirus capsid were induced by the human chimeric strain, we next performed serum neutralisation assays. Sera from 10-week-old mice were incubated with Rotarix for 1 h, and then the resulting complexes were added to MA104 cells and infection was allowed to proceed overnight (Figure 3F). Whereas sera from mice infected with entirely murine chimeric virus could neutralise to a mean of 30.0% relative to the no-sera control, sera from mice infected with virus containing human outer capsid proteins neutralised to 14.2% (*p* < 0.0001). This provides clear evidence that a human-outer-capsid-specific antibody response was induced by this chimeric virus.

To verify that this functional response was specific for the outer capsid and not for other rotavirus proteins, we also performed an intracellular neutralisation assay. This assay evaluates the activity of VP6-specific antibodies inside cells, achieved when serum antibodies are electroporated into the cytoplasm of MA104 cells [13]. As the VP6 sequence was identical for both chimeric viruses, we hypothesised that intracellular neutralisation by sera from infected mice would be very similar. As shown in Figure 3G, there was indeed no significant difference in intracellular neutralisation induced by the antibodies raised to both chimeric viruses.

### 3.4. Chimeric Viruses Induced Human-Rotavirus-Specific B Cell Responses in Mice

**Figure 4:**
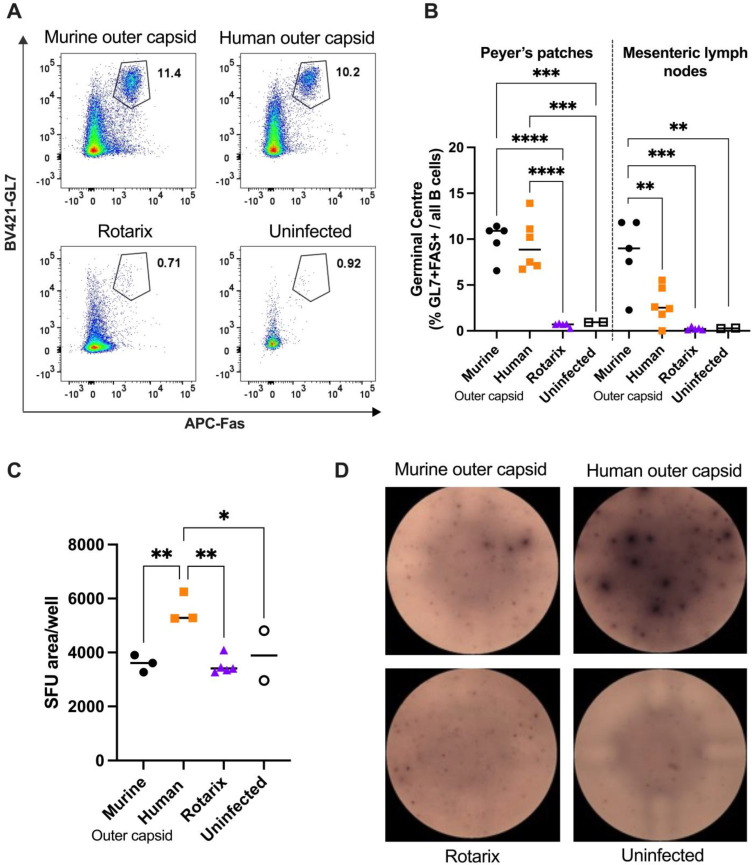
Analysis of B cell responses in mice infected with chimeric (murine outer capsid as black circles, human outer capsid as orange squares) or human Rotarix control (shown as purple triangles). Seven-day-old mice were infected with the panel of viruses, and B cell analysis was performed 14 days later. (**A**) Representative flow plots of germinal centre (GL7 + FAS+) B cells identified in Peyer’s patches (PPs) by flow cytometry. Numbers represent the percentage of total B220 + cells. (**B**) Quantification of germinal centres in PPs and MLNs by flow cytometry. (**C**) Quantification of spot forming unit (SFU) area by B cell ELISpot for Rotarix-specific B cells. (**D**) Representative images of B cell ELISpot wells. Statistical significance was determined by one-way ANOVA. Tukey’s adjusted pair-wise comparisons are shown for *p* < 0.05 (* *p* < 0.05; ** *p* < 0.01; *** *p* < 0.001; **** *p* < 0.0001).

To complement and extend the results obtained from the serum antibody analysis of mice infected with chimeric viruses, we also characterised B cell responses to infection. Two litters of mice were infected with chimeric viruses, and 14 days post-infection, cells from the spleen and draining lymph nodes (PPs and MLNs) were analysed. A third litter of mice was infected with an equal titre of the Rotarix vaccine strain to verify the inability of an entirely human rotavirus strain to induce a detectable B cell response in mice. Two age-matched uninfected mice were also included as controls. Flow cytometry on 50,000 cells was used to identify the germinal centre B cells present in the PPs and MLNs of each litter of mice (Figure 4A,B). No germinal centre formation was observed in pups infected with Rotarix, in accordance with Figure 3A, and in line with the lack of virus replication measured in Figure 2A. In contrast, germinal centre formation in draining lymph nodes was readily observed in both litters of mice infected with the two chimeric rotavirus strains. Interestingly, there was a small but significant difference (*p* = 0.0128) between the number of germinal centre B cells measured in the MLNs. This indicates that the chimeric strain with the human rotavirus outer capsid induces fewer germinal centre B cells than the entirely murine strain.

Whilst germinal centre B cell quantification clearly shows a strong B cell response, this approach does not identify antigen specificity of the B cells. To investigate this, we performed ELISpot assays, using Rotarix as the antigen coated onto wells of an ELISpot plate. Splenocytes from three mice from each litter, plus from two uninfected control mice, were incubated in the antigen-coated wells overnight, then staining for IgG production was completed the following day. Figure 4C,D show that mice infected with chimeric virus encoding the human outer capsid generated B cells that produced significantly more human-rotavirus-specific antibodies than the other viruses. This therefore confirms that a human-rotavirus-specific B cell response can be readily induced and detected by infection with a chimeric virus.

## 4. Discussion

The species-specificity of rotaviruses has made development of a small animal model to study human rotavirus strains a significant challenge. This has hampered efforts to characterise the immune responses to human rotavirus by experimental infections, and means that a robust pre-clinical system to analyse the efficacy of vaccine candidates and therapeutics has been lacking. To address this, we have successfully generated a novel approach to permit replication of rotaviruses encoding human rotavirus proteins in mice, and shown that these mice generate robust antibody responses to human rotavirus proteins. This was achieved using reverse genetics to create a chimeric rotavirus that encodes the outer capsid proteins of a human rotavirus. We verified that whereas human rotavirus stains replicate poorly in mice and are not immunogenic, a chimeric rotavirus replicates to a high titre and enables characterisation and quantification of human-rotavirus-outer-capsid-specific antibody responses. These viruses could also facilitate future analysis of additional immune responses to human outer capsid proteins, including B cell memory induction and VP4- and VP7-specific T cells.

A common alternative approach to studying non-murine viruses in mouse models is to use immunocompromised mice [15,16]. This has been used to permit replication of non-murine rotaviruses in mouse models, e.g., STAT1 knockout mice [6,17], but this does not provide a comprehensive overview of the interactions between the innate and adaptive immune responses. Therefore, use of our chimeric virus system has an advantage over the use of immunocompromised mice, as an immune response that more closely resembles the complexity and functionality seen in humans is induced. This generates a more representative picture of the multifaceted immune response to the outer capsid epitopes that occurs in a human rotavirus infection. It is acknowledged that the use of humanised mice could be a means of advancing this model further, but use of the widely available wild-type 129S6/SvEvTac mouse strain makes our approach more accessible.

We propose a number of different situations where this chimeric rotavirus could be valuable for advancing our understanding of how to control human rotavirus infections. Firstly, the ability to study antibody responses to human rotavirus outer capsid proteins could be valuable for investigating a number of factors that have been associated with reduced rotavirus vaccine efficacy in low- and middle-income countries. For example, maternal antibodies have been correlated with a reduced ability of infants to seroconvert following vaccination in a number of vaccine clinical trials, yet the target of these interfering maternal antibodies is unclear [18,19]. Infection of female mice with one strain, and then infection of their pups with another would determine whether antibodies targeting the outer capsid protein are responsible for interference. A second situation where these viruses could be useful is in pre-clinical vaccine trials. Whereas immune responses to mice vaccinated with new strategies targeting human rotaviruses, e.g., recently described rotavirus VP8 * mRNA vaccines [20], can be readily studied in mice, the ability of these immune responses to protect against human rotavirus infection is not possible using standard strains. A chimeric virus infection would provide a solution for this. Finally, chimeric viruses could be used to test new therapeutic strategies targeting the human outer capsid protein. This would be especially useful for testing monoclonal antibodies specific for human rotavirus strains [21,22].

One potential limitation of our model is the inability of the human chimeric virus to recapitulate the gastrointestinal disease seen in natural species-specific rotavirus infections. Current rotavirus vaccines do not induce sterilising immunity, but instead reduce the severity of clinical signs following infection. The ideal model system would therefore induce gastroenteritis in mice in order to enable testing of vaccine candidates or therapeutics that aim to reduce the severity of clinical disease. Whilst the viral replication in mice infected with rotaviruses with murine or human outer capsid proteins was similar, there was an interesting decrease in pathogenicity when switching from mouse to human. We found that the incidence of diarrhoea in pups infected with the rotavirus with a human capsid was significantly reduced. It is known that the pathogenesis of diarrhoea in rotavirus infection is multi-factorial, with reduced epithelial absorption, NSP4 enterotoxins, and activation of the nervous system all reported to be involved [23]. As the only difference between our two chimeric viruses was the outer capsid protein, this eliminates a possible role for NSP4, and instead suggests that the interaction of the outer capsid proteins and the murine intestinal tract is important. We hypothesise that differences in outer capsid proteins alter the region of the intestines where the virus preferentially binds and replicates. This could be further explored by immunohistochemistry of the entire intestinal tract post-infection.

An additional limitation of our outer capsid chimera approach is that this model does not enable study of a human-rotavirus-VP6-specific immune response. This is an issue, as the middle capsid protein VP6 is highly immunogenic and known to be the target of many rotavirus-specific antibodies in humans [24,25]. Furthermore, VP6-specific antibodies have been shown to be protective in mouse models [13,26]. The absence of human-rotavirus-specific VP6 in our chimeric virus system means the repertoire of antibodies induced by chimeric viruses will not fully recapitulate that induced by natural human rotavirus strains. Similarly, any T cell responses to human strain VP6 will not be evident. One possible solution to this issue could be to generate a chimeric rotavirus with human VP4, VP7, and VP6 on a murine backbone. However, this is predicted to be technically challenging, as VP6 plays a crucial role in the structure and function of rotaviruses, and therefore a human strain VP6 may not be compatible with a murine rotavirus backbone.

## 5. Conclusions

In conclusion, we have shown that using reverse genetics to manipulate rotaviruses can be an effective strategy to study human rotaviruses in an immunocompetent pre-clinical model. This approach has the potential to facilitate future vaccine and therapeutic development against a childhood pathogen whose global disease burden is still unacceptably high.

## Figures and Tables

**Table 1:** Reagent details.

Reagent	Source	Identifier
Antibodies and Dyes		
Alexa Fluor 488 Anti-Sheep IgG (Donkey)	Invitrogen, Carlsbad, CA, USA	CAT#A-11015
Anti-Mouse IgG Biotin (Goat)	Mabtech, Cincinnati, OH, USA	CAT#3825-6
Anti-Mouse IgG HRP (Goat)	Sigma Aldrich, St. Louis, MO, USA	CAT#A0168
CD45 (BUV395 Rat Anti-Mouse)	BD Biosciences, San Jose, CA, USA	CAT#564279; Clone: 30-F11
CD45R (PerCP/Cyanine 5.5 Anti-Mouse/Human CD45R/B220)	BioLegend, San Diego, CA, USA	CAT#103236; Clone: RA3-6B2
CD95 (APC Anti-Mouse (Fas))	BioLegend, San Diego, CA, USA	CAT#152603; Clone: SA367H8
Clarity Western ECL Substrate	Bio-Rad, Hercules, CA, USA	CAT#170-5060S
Fc Block (TruStain FcX Anti-Mouse CD16/32)	BioLegend, San Diego, CA, USA	CAT#101320; Clone: 93
Fixable Viability Dye eFluor 780	Invitrogen, Carlsbad, CA, USA	CAT#65-0865
GL7 Antigen (Pacific Blue Anti-Mouse/Human)	BioLegend, San Diego, CA, USA	CAT#144614; Clone: GL7
Hoechst 33342	Invitrogen, Carlsbad, CA, USA	CAT#H3570
Rotavirus Polyclonal Antibody (Sheep)	Invitrogen, Carlsbad, CA, USA	CAT#PA1-85845
Streptavidin–ALP	Mabtech, Cincinnati, OH, USA	CAT#3310-10
Chemicals		
BCIP/NBT-plus for ALP	Mabtech, Cincinnati, OH, USA	CAT#3650-10
Dulbecco’s Modified Eagle’s Medium (DMEM)	Corning, Corning, NY, USA	CAT#10-013
Fetal Bovine Serum (FBS)	Corning, Corning, NY, USA	CAT#28622001
Laemmli Sample Buffer (4×)	Bio-Rad, Hercules, CA, USA	CAT#1610747
Pierce Protease Inhibitor Mini Tablets	Thermo Fisher Scientific, Waltham, MA, USA	CAT#A32953
Red Blood Cell Lysis Buffer (10×)	BioLegend, San Diego, CA, USA	CAT#420301
RIPA Lysis and Extraction Buffer	Thermo Fisher Scientific, Waltham, MA, USA	CAT#89900
RPMI 1640	Corning, Corning, NY, USA	CAT#10-040-CV
TPCK-Treated Trypsin	Worthington Biochemical, Lakewood, NJ, USA	CAT#LS003740
3,3’,5,5’-Tetramethylbenzidine (TMB) Membrane Peroxidase Substrate Plus	Avantor, Radnor, PA, USA	CAT#K830
Commercial Assays		
BCA Protein Assay Kit	Thermo Fisher Scientific, Waltham, MA, USA	CAT#23227
Luna Universal One-Step RT-qPCR Kit	New England Biolabs, Ipswich, MA, USA	CAT#E3006
Monarch Total RNA Miniprep Kit	New England Biolabs, Ipswich, MA, USA	CAT#T2010S
Neon Transfection System 100 μL Kit	Invitrogen, Carlsbad, CA, USA	CAT#MPK10025

## Data Availability

All the relevant data are provided in this paper.
